# An In Vitro Model of Gastric Inflammation and Treatment with Cobalamin

**DOI:** 10.1155/2017/5968618

**Published:** 2017-06-06

**Authors:** T. R. Elliott, A. L. Guildford

**Affiliations:** Brighton Studies in Tissue-Mimicry and Aided Regeneration (BrightSTAR), Brighton Centre for Regenerative Medicine, School of Pharmacy and Biomolecular Sciences, University of Brighton, Lewes Road, Brighton BN2 4GJ, UK

## Abstract

Pernicious anaemia (PA) is an autoimmune condition where antibodies target intrinsic factor and parietal cells, reducing the patient's ability to absorb cobalamin promoting atrophic gastritis. Treatment guidelines are based on excretion data of hydroxocobalamin from healthy individuals obtained 50 years ago. This manuscript describes the use of phorbol 12-myristate 13-acetate (PMA) to stimulate low grade inflammation in an epithelial colorectal cell line to assess the efficacy of methylcobalamin and hydroxocobalamin. Nitric oxide increased significantly in cells exposed to higher doses of PMA (100 ng/ml, 150 ng/ml, and 200 ng/ml) accompanied by a loss of the characteristic cobblestone morphology with no negative effect on cell activity or viability. A significant reduction in nitric oxide production was associated with the addition of 200 pg/ml hydroxocobalamin, alongside a return to the characteristic cobblestone morphology. This study highlights the use of PMA to promote low grade inflammation in human cell lines to model gastric inflammation associated with autoimmunity; furthermore it raises questions regarding the concentration of cobalamin administered clinically to restore cell functionality, feasibly allowing the patient to receive reduced quantity of the vitamin more regularly, providing the patient with levels which are akin to dietary intake.

## 1. Introduction

Patients with inflammatory bowel conditions often suffer from cobalamin deficiency [1]. It can also lead to pernicious anaemia (PA) an autoimmune condition associated with atrophic gastritis resulting in further gastric inflammation. In atrophic gastritis the parietal cells [2] are destroyed by autoantibodies towards the H^+^K^+^-ATPase on the surface of the cell [3]. This destruction leads to the decreased secretion of intrinsic factor (IF), a glycoprotein [4] essential for the absorption of cobalamin [5]. Oral B12 has been trialled; however only 8% of UK patients surveyed were on oral or sublingual medication [6]. Intramuscular injection is the most frequently administered form of treatment. Untreated cobalamin deficiency causes psychological symptoms such as depression, anxiety, and Alzheimer's disease. Subacute cerebral degeneration is a severe but frequently seen symptom which presents as general weakness, paraesthesia, abnormal gait, stiffness, and limb weakness [7].

Cobalamin is an essential water soluble vitamin [8] required as a coenzyme for methionine synthase and methylmalonyl-CoA mutase [4], in the methylation of DNA and the Krebs cycle, respectively. Cobalamin is an organometallic compound with a corrin ring structure surrounding a cobalt molecule [9]. It cannot be synthesized by the body and must be taken in through the diet. Cobalamin exists in four forms: hydroxocobalamin, methylcobalamin, cyanocobalamin, and adenosylcobalamin. Methylcobalamin and adenosylcobalamin are biologically active forms of cobalamin [10] whereas hydroxocobalamin and cyanocobalamin are inactive. In previous studies the active forms of cobalamin were found to be protective against cell death induced by increased homocysteine [11]. Elevated homocysteine levels have been previously linked with an increased risk of myocardial infarction and Alzheimer's disease. Methylcobalamin, acting as a coenzyme with methionine synthase, converts homocysteine to methionine. Therefore it is not surprising that 98% of patients with cobalamin deficiency have an increased total homocysteine level [12].

The recommended treatment for cobalamin deficiency is injections of cobalamin. In the UK patients receive subcutaneous injections of hydroxocobalamin every 12 weeks [6]; however 64% of participants of a survey by the PA Society (UK) were unhappy with their treatment [6] due to symptom recurrence before their next injection. It is essential that patients receive effective and appropriate treatment for the rest of their lives to ensure a good a quality of life. The current BNF treatment regime in the UK was based on a 1968 paper [7] which studied cobalamin excretion. They found the inactive hydroxocobalamin was retained for longer in the body than the active cyanocobalamin. However, the paper cohort was small, 13 in total, and none of the participants were diagnosed with PA, B12 deficiency, or associated transport problems. To our knowledge, no further research has been undertaken in subjects with PA or associated gastritis to understand the impact of the disease on cobalamin excretion or more importantly its direct cellular effect.

Nitric oxide (NO) has been implicated in gastric inflammation, along with NO synthase (NOS). These have been shown to contribute to the pathology of inflammation by causing cell cytotoxicity [13]. Cobalamin has been shown to reduce the bioactivity of NO, as NO will bind to the corrin ring structure of cobalamin [14]. A 2009 study found that hydroxocobalamin was the most effective form of cobalamin in inhibiting NOS [14].

Caco-2 cells are a human colon adenocarcinoma cell line that has been used to model the gastric epithelium for almost 30 years [15]. In vitro models using Caco-2 cells to mimic gastric inflammation [16] in coeliac disease found that a combination of phorbol myristate acetate (PMA) and IFN-*γ* was the best inducer of NO production and therefore inflammation. Another study found that PMA alone was sufficient to induce inflammation in Caco-2 cells though at a lower level compared to IFN-*γ* [15]. In this experimental model inflammation will be induced using PMA alone to mimic a low level of acute gastric inflammation.

The aim of this study is to develop an in vitro model capable of mimicking low grade gastric inflammation and to use this model to investigate the activity of clinically relevant levels of cobalamin as defined by earlier studies and finally to enhance our current understanding between the impact of active and inactive cobalamin in the developed gastric model of inflammation.

## 2. Materials and Methods

### 2.1. Gastric Inflammation Study

Caco-2 cells were subcultured at a subcultivation ratio of 1 : 5 in DMEM 10% FBS (37°C, 95% O_2_, 5% CO_2_); 1 × 10^5^ cells were seeded in to 24-well plates and allowed to adhere for 24 hours. Cells were spiked with phorbol 12-myristate 13-acetate (PMA) (0, 50, 100, 150, and 200 ng/ml) and incubated for 24 and 48 hours. After incubation media were removed and centrifuged, processed for the Griess assay and MTT assays, respectively.

#### 2.1.1. NO Production

Griess assay (Promega, UK): sulfanilamide solution was added to 50 *μ*l of each sample and incubated in the dark prior to the addition of N-(1-naphthyl)ethylenediamine dihydrochloride (NED). After incubation the absorbance was read at 520 nm. The limit of detection is 2.5 *μ*M (125 pmol) nitrite.

#### 2.1.2. Cell Activity

MTT (Sigma-Aldrich, UK) was added to the remaining wells and incubated; formazan crystal in wells was dissolved using MTT solubilization solution and the absorbance measured at 570 nm.

#### 2.1.3. Cell Viability and Morphology

5-Chloromethylfluorescein diacetate in DMSO (10 *μ*M) (Sigma) was added to wells and incubated with media (DMEM + 10% FBS) prior to fixing in formalin (3.7%) and observing under the confocal microscope (Leica, UK).

### 2.2. Active Cobalamin Toxicity Study

Caco-2 cells were seeded at 1 × 10^5^ and allowed to adhere for 24 hours. Cells were spiked with methylcobalamin at clinically relevant concentrations (Table 1) and incubated for 24 hours. Supernatant and cells were processed as above for NO production (Section 2.1.1) and cell viability using MTT (Section 2.1.2).

### 2.3. In Vitro Model Study

Caco-2 cells were seeded (1 × 10^5^) to 24-well plates and allowed to adhere for 24 hours in DMEM 10% FBS (37°C, 95% O_2_, and 5% CO_2_). Cells were spiked with 150 ng/ml phorbol-12-myristate 13-acetate and methylcobalamin (0, 200, 500, 750, and 1000 pg/ml) or hydroxocobalamin (0, 200, 500, 750, and 1000 pg/ml) and incubated for 24 hours at 37°C, 5% CO_2_. Supernatants and cells were processed for NO production using the Griess assay (Section 2.1.1) and cell viability via MTT assay (Section 2.1.2). Cell morphology was observed using phalloidin and rhodamine staining; the cells were fixed in 3.7% formalin prior to washing in sterile PBS and incubation in phalloidin rhodamine; after incubation the dye was removed and the samples were viewed under the confocal microscope.

### 2.4. Statistics

All statistical information was determined using one-way ANOVA and Tukey's method and 95% confidence intervals using Minitab Statistical software.

## 3. Results

### 3.1. Gastric Inflammation Study

#### 3.1.1. Nitric Oxide Production

**Figure 1:**
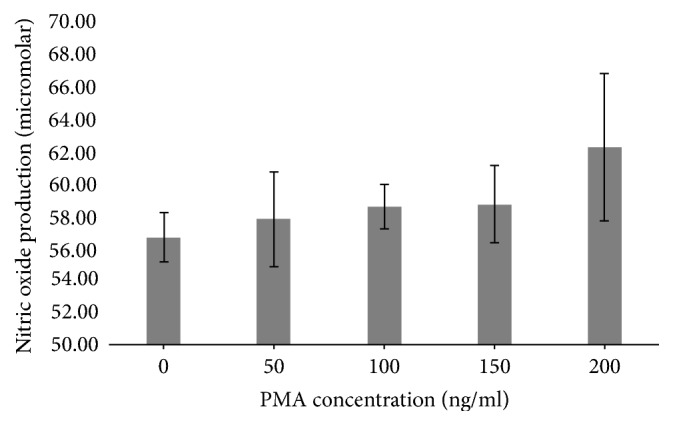
Nitric oxide production from colorectal adenocarcinoma (Caco-2) cells after 24-hour culture with phorbol 12-myristate 13-acetate (PMA).

Caco-2 cells incubated with PMA showed a positive significant increase in cell production of nitric oxide with 100, 150, and 200 ng/ml compared to cells incubated with 0 and 50 ng/ml PMA (*p* = 0.0038) (Figure 1).

#### 3.1.2. Cell Activity

**Figure 2:**
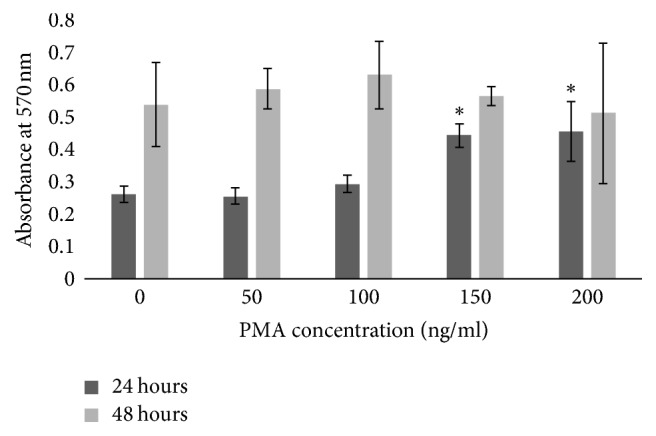
Cell metabolic activity as measured by MTT assay after 24-hour and 48-hour incubation in culture with phorbol 12-myristate 13-acetate (PMA) (^*∗*^significantly increased (*p* = 0.0013)).

Cells incubated with PMA for 24 and 48 hours showed an increase in cell activity. Cells incubated for 24 hours demonstrated a significant increase in cell activity from 100 ng/ml reaching a peak at 150 and 200 ng/ml, where the absorbance for cell activity was significantly greater than at 0, 50, and 100 ng/ml (*p* = 0.0013). Cells at 48 hours demonstrated the greatest activity; indeed there was a significant increase in cell activity of all cells incubated for 48 hours (*p* = 0.7840); however the activity had plateaued and increasing the concentration of PMA did not influence cell activity (Figure 2).

#### 3.1.3. Cell Viability and Morphology

**Figure 3:**
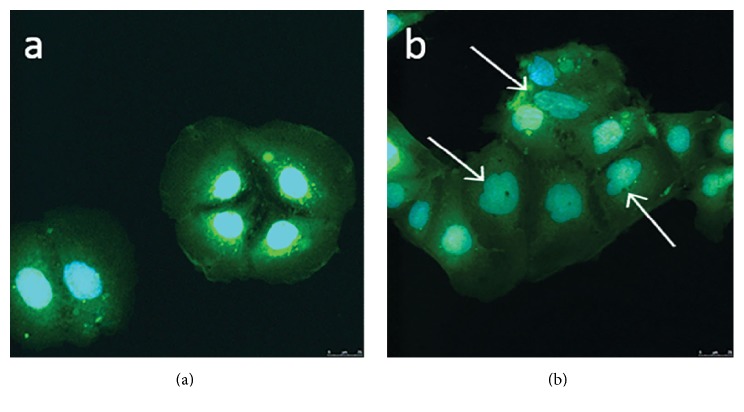
Colorectal adenocarcinoma (Caco-2) cells after 24-hour incubation with (a) cell culture medium and (b) cell culture medium spiked with 150 ng/ml phorbol 12-myristate 13-acetate (PMA). White arrows highlight nuclear changes.

Morphological changes were observed between the control cells incubated without PMA and those incubated with 150 ng/ml of PMA and above over 24 hours. Control Caco-2 cells demonstrated a typical cobblestone morphology (Figure 3(a)), where the cells were of a similar size with an ovular shaped nucleus. In contrast, cells with 150 ng/ml PMA displayed an altered morphological appearance; the cells are clustered but have lost the stereotypical cobblestone morphology and regular nucleus, and rather the cells show a more atypical nucleus (Figure 3(b) white arrows) with identifiable filopodia and extrusions (data not shown).

### 3.2. Active Cobalamin Toxicity Study

#### 3.2.1. NO Production

**Figure 4:**
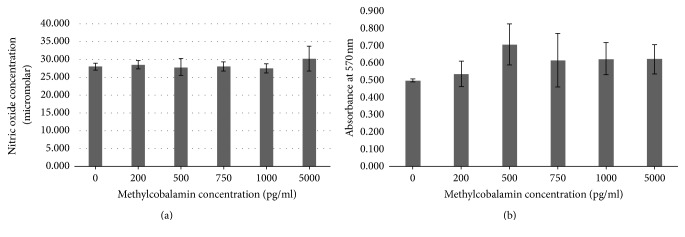
Colorectal adenocarcinoma (Caco-2) cells after 24-hour culture with methylcobalamin (0, 200, 500, 750, 1000, and 5000 pg·ml). (a) Nitric oxide production; (b) cell activity as measured by MTT.

Cells incubated methylcobalamin demonstrated no significant changes in NO release compared to controls, and levels of the active cobalamin which reached a fivefold excess of clinical standards showed no significant difference in the level of NO production (*p* = 0.1472) (Figure 4(a)).

#### 3.2.2. Cell Activity

There was no significant statistical difference in Caco-2 cell activity after 24-hour incubation with different concentrations of methylcobalamin (*p* = 0.3118) (Figure 4(b)) (*p* = 0.3118).

### 3.3. In Vitro Model Study

#### 3.3.1. Methylcobalamin

**Figure 5:**
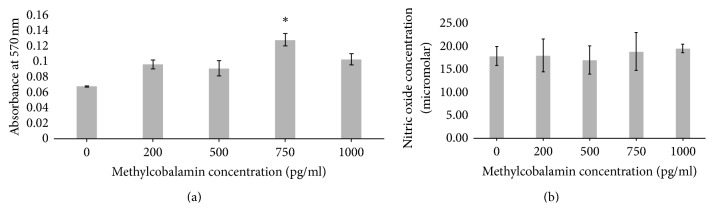
Colorectal adenocarcinoma (Caco-2) cells after 24-hour culture with 150 ng/ml phorbol 12-myristate 13-acetate (PMA) and methylcobalamin (0, 200, 500, 750, and 1000 pg/ml). (a) Cell activity as measured by MTT; (b) nitric oxide production (^*∗*^significantly increased *p* = 0.0061).

**Figure 6:**
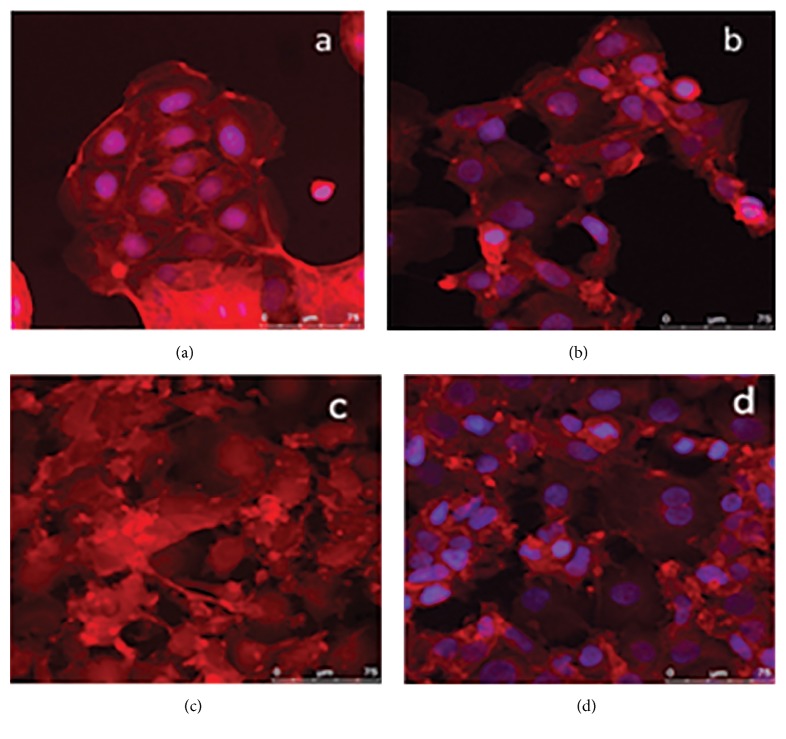
Colorectal adenocarcinoma (Caco-2) cells after 24-hour culture with (a) 150 ng/ml phorbol 12-myristate 13-acetate (PMA) (b) and 200 pg/ml methylcobalamin (c) or 1000 pg/ml methylcobalamin (d).

There is a significant increase in cell activity with 750 pg/ml of methylcobalamin with PMA compared to all other concentrations of methylcobalamin (*p* = 0.0061) (Figure 5(a)). There is no significant statistical difference in NO production between cells with and without PMA at the same concentrations of methylcobalamin (Figure 5(b)) (*p* = 0.1839). The morphology of the cell (Figure 6) shows distinct changes from cells incubated with the addition of methylcobalamin. When incubated with the lowest clinical value of methylcobalamin 200 pg/ml (Figure 6(c)) the cells are elongated rather than cobblestone; they have a reduced cytoplasm and a rougher cell surface and they appear to have lost their ability to form a monolayer in comparison to the control Caco-2 cells (Figure 6(a)). Indeed, their overall appearance looks similar to that of the cells in the PMA control (Figure 6(b)) where the rougher cell surface is also associated with a change of the shape of the nucleus and a tendency towards more spiked cell morphology. The cells incubated with the highest dose of methylcobalamin 1000 pg/ml showed a mixed cell population where those on the tissue culture plastic demonstrated a flatter more rounded morphology, with a large round nucleus and a cytoplasm ratio akin to that of the cell control (Figure 6(a)) but these unlike the cell control were covered with a number of cells more representative of the PMA control with a more irregular nucleus and a reduction in cytoplasm (Figure 6(b)).

#### 3.3.2. Hydroxocobalamin

**Figure 7:**
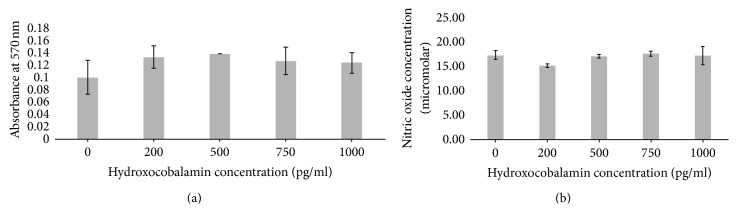
Colorectal adenocarcinoma (Caco-2) cells after 24-hour culture with 150 ng/ml phorbol 12-myristate 13-acetate (PMA) and hydroxocobalamin (0, 200, 500, 750, and 1000 pg/ml): (a) cell activity as measured by MTT; (b) nitric oxide production.

**Figure 8:**
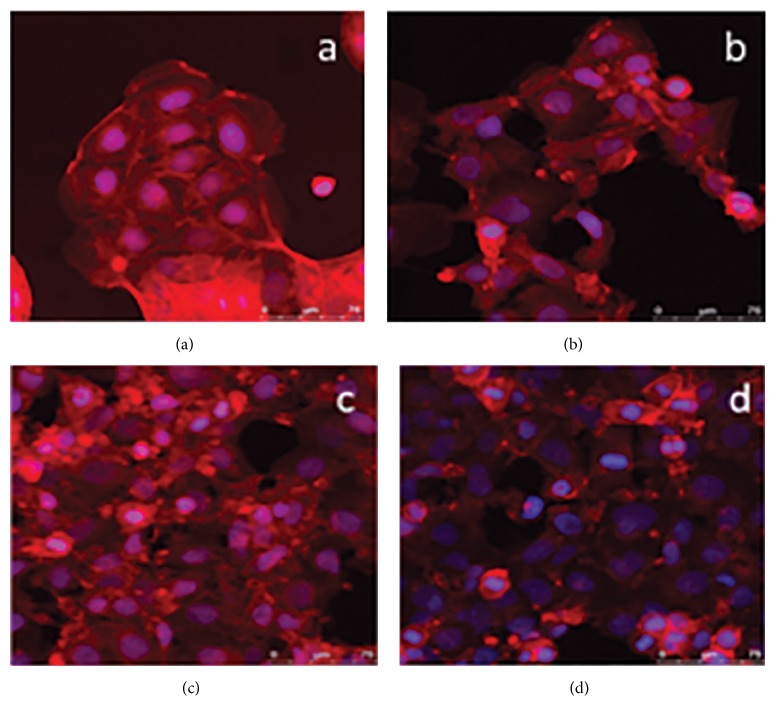
Colorectal adenocarcinoma (Caco-2) cells after 24-hour culture with (a) 150 ng/ml phorbol 12-myristate 13-acetate (PMA) (b) and 150 pg/ml hydroxocobalamin (c) or 1000 pg/ml hydroxocobalamin (d).

No significant difference was observed in cell activity with PMA (*p* = 0.1941) (Figure 7(a)). However, NO production was significantly reduced with the addition of 200 pg/ml hydroxocobalamin (Figure 7(b)) (*p* = 0.0008). Imaging of cell morphology highlighted a clear morphological change in Caco-2 cells incubated with PMA and hydroxocobalamin (Figure 8). As described cells incubated with PMA demonstrate an irregular nucleus and a lack of typical cobblestone morphology (Figure 8(b)), displaying filopodia extensions. Cells incubated with 150 ng/ml hydroxocobalamin start to demonstrate features of both populations with cells demonstrating regular nuclei and fewer projections (Figure 8(c)). Cells further resemble the controls cells when exposed to 1000 pg/ml hydroxocobalamin (Figure 8(d)), the cell shape and size having become regular; however the cells have not yet formed clear clusters seen in control cells (Figure 8(a)).

## 4. Discussion

For this study, PMA was used as the inducer of inflammation as described by Chen and Kitts [15]. PMA activates protein kinase C (PKC) in epithelial cells [17] by mimicking diacylglycerol, a natural ligand to PKC. It has been used to promote inflammation both in vitro [15, 16, 18] and in vivo [19]. 200 mg/ml PMA showed the greatest increase in NO production (Figure 1) along with the greatest increase in cell activity (Figure 2). However, a distinct increase in both cell activity and NO production was seen from concentrations as low as 100 ng/ml of PMA. Cell morphology showed clear alterations from the formation of cell clusters with a smooth cobblestone appearance to rougher surface morphology where the individual cell nucleus was larger and the cells were less uniform with the addition of PMA (Figure 3). Similar morphological changes have been observed in other studies, where cells incubated with PMA and IFN-*γ* showed changes when viewed using phase contrast microscopy [16]. Due to the insidious nature of inflammatory disease a midrange activation concentration of 150 ng/ml of PMA was chosen for the model as this dose generated a significant increase in cell activity alongside moderate increase in NO production which can be associated with low grade inflammation.

For the toxicity study, we used the biologically active form of the vitamin, methylcobalamin, because it can be used as a coenzyme by the cell without conversion. Clinically relevant doses were used in this study to reflect current treatment regimes. The methylcobalamin did not show any significant increase in cell activity (Figure 4(a)) or in NO production (Figure 4(b)). These data confirm the lack of gastric epithelial cell toxicity of methylcobalamin which was used up to values five times greater than the upper limit of cobalamin (5000 pg/ml). This high concentration and lack of toxicity are supported by clinical trials in Japan, where patients with peripheral neuropathy were treated with 25 mg/day IV methylcobalamin [20] over 10 days for five months. This is 2.5 × 10^10^ times greater than the recommended dose for patients with PA who are given 1 mg/ml every 8–12 weeks. These high doses showed no adverse side effects to patients, which is supported by our in vitro model. Similarly, high concentrations of inactive cobalamin are used clinically to treat cyanide poisoning at very high doses of 4-5 g repeated over consecutive days [11]. Reported side effect were only as a transient urticarial rash; therefore toxicity is minimal with extremely high doses over the short term.

The in vitro model was developed to observe the impact of the atrophic gastritis on the cells of the gastric epithelium, as the action of the cells can be linked to the progression and development of the disease. In this model inflammation was achieved by the addition of 150 ng/ml PMA. This concentration was identified as it generated an increase in NO production from the cell alongside cell morphology alterations, both of which are proposed to reflect a change in function of the cell associated with low grade inflammation, as identified clinically in patients suffering from autoimmune conditions. To observe the effect of the different cobalamins, clinically relevant doses were incubated with the model.

Change in morphology lends itself to a change in function; individual Caco-2 cells observed under inflamed conditions in this model separated from the continuous monolayer associated with a fully functional gastric lining, therefore losing cell-cell tight junctions as seen in normal culture. The lack of continuous monolayer and loss of tight junctions can themselves act as an inflammatory trigger exacerbating the situation, potentially leading to chronic inflammation associated with comorbid conditions such as gastric cancer. Similarly ultrastructural alterations have also been implicated in altered cell proliferation and cellular kinetics both of which will impact the structure, function, and integrity of the epithelial lining [22].

The cells in this model demonstrated the ability to return to more typical morphology postinflammatory stimuli after incubation with higher levels of cobalamin, giving a more clinically reflective model of acute pathological conditions characterised by excessive NO production in the intestine, which leads to the increased permeability of cells, a potential trigger of inflammatory disease [20].

Methylcobalamin showed no observable differences towards the suppression of NO (Figure 5); however this may have been masked by the increased cell activity observed at higher concentrations in the presence of inflammatory stimuli (Figure 5).

This could be due to the availability of the biologically active form for inclusion into the normal cell processes of DNA methylation and the Krebs cycle as postulated [1]. It may also be that the upregulation of these processes has prevented the availability of the methylcobalamin for the purpose of NO inhibition. This implies a prioritisation of basic cell function over its protective role. Despite the lack of observable NO inhibition, morphological studies highlighted the return of the cell morphology to be more reminiscent of the control cells on the addition of the biologically active cobalamin in the presence of the inflammatory stimuli.

By contrast the inactive hydroxocobalamin showed no observable increase in cell activity (Figure 6(b)), combined with a trend towards a reduction in the amount of freely available NO under inflammatory stimuli. Indeed, there is a significant difference between cells incubated with PMA and hydroxocobalamin at 200 and 500 pg/ml and their respective counterparts without PMA. The biologically inactive nature of hydroxocobalamin may prevent its immediate uptake into cellular processes as it needs to be converted to methylcobalamin before it can be used by the cells [17], allowing it to remain available for the “mopping” up of endogenous NO. Cellular pretreatment with inactive cobalamin has been noted in previous studies as preventing the process of apoptosis [23].

The reduction in NO concentration with the addition of cobalamin was highlighted but not significant in these studies; it is hypothesised that this is due to the mimicking of a low grade inflammatory condition. Birch et al. [24] further demonstrated the antioxidant potential of cobalamin conferred by its ability to reduce NO concentrations. Similarly, studies have also shown an increase in the transporter protein transcobalamin in acute and chronic inflammatory states [11].

Methylcobalamin serves to reduce homocysteine to methionine via methionine synthase, which alongside ATP ensures the methylation of RNA, DNA, and proteins linked with endothelial cell dysfunction and the formation of reactive oxygen species (ROS) including NOS [11]. As a result, patients with cobalamin deficiency possess a decreased ability to inhibit NO alongside an increased NO production, promoting a perpetual inflammatory state with systemic clinical impact. Indeed, increased homocysteine levels are associated with the induction of apoptosis in human bone marrow stromal cells, human umbilical vein endothelial cells, and endothelial progenitor cells; the apoptosis was shown to be prevented by the pretreatment with inactive cobalamin metabolism [21].

The concentration range of clinically acceptable plasma cobalamin varies between countries. In the UK, patients are considered borderline abnormal when the cobalamin levels are between 120 and 180 pg/ml and below 120 pg/ml is an abnormal result [12]. In Asian countries the lower limit is 500 pg/ml [21]. This discrepancy means patients who are considered normal in European countries would be considered as having a low cobalamin elsewhere, and so patients with cobalamin deficiency will be missing out on treatment, leading to further deterioration of their condition before diagnosis causing great distress for the patient. In this study, low concentrations of cobalamin (200 pg/ml) benefitted gastric epithelial cells in an inflammatory environment by restoring some of the features of normal gastric cell morphology; however the cells still remained fairly atypical in shape and size. It can be argued that patients presenting with symptoms and cobalamin levels less than 500 pg/ml should receive cobalamin treatment as the levels in the body may not be sufficient to reduce excessive inflammation. This would move the UK lower limit of normal in line with that of Asia.

The recommended treatment of cobalamin deficiency is not sufficient for a large number of patients [8]; its symptoms are systemic as a result of the importance of cobalamin in DNA replication and respiration. This study demonstrated that PMA is a suitable inducer of low grade gastric inflammation; it highlights that different forms of cobalamin may be more suitable for treatment due to their ability to be taken up with cellular processes. This work has highlighted that levels below those currently administered promote a beneficial cellular effect; overall more investigation is needed to define to optimal treatment approach based on cellular activity and health, not defined by the systemic tolerance of healthy individuals.

## Figures and Tables

**Table 1:** Cobalamin concentration and the clinical relevance.

Cobalamin concentration (pg/ml)	Clinical relevance of value
0	Control value
200	UK minimum of normal range [21]
500	Asian minimum of normal range [22]
750	Midrange of reference range
1000	UK maximum of normal range [21]
5000	Five times greater than maximum range to test for toxicity of extremes
